# Study of the Factors Involved in the Adhesion Process of *Salmonella enterica* Enteritidis, *Escherichia coli*, and *Staphylococcus aureus* to the Surface of Apple, Arugula, Cucumber, and Strawberry

**DOI:** 10.3390/foods15030449

**Published:** 2026-01-27

**Authors:** Jéssica Souza Rocha, Bárbara Morandi Lepaus, Manueli Monciozo Domingos, Patrícia Campos Bernardes, Jackline Freitas Brilhante de São José

**Affiliations:** 1 Integrated Health Education Department, Federal University of Espírito Santo, Vitória 29040-090, ES, Brazil; jessicasouzar@hotmail.com; 2 Postgraduate Program in Nutrition and Health, Federal University of Espírito Santo, Vitória 29040-090, ES, Brazil; 3 Department of Food Engineering, Federal University of Espírito Santo, Alegre 29500-000, ES, Brazil

**Keywords:** fresh produce safety, bacterial attachment, surface roughness, hydrophobicity, temperature control, thermodynamic prediction

## Abstract

Bacterial contamination of fresh produce remains a global food safety concern, with pathogens such as *Salmonella*, *Escherichia coli*, and *Staphylococcus aureus* frequently implicated in foodborne outbreaks. Understanding the physicochemical factors involved in bacterial adhesion to fresh produce surfaces is essential for developing effective sanitization strategies. This study evaluated the influence of surface roughness, hydrophobicity, thermodynamic free energy, and temperature on pathogen adhesion to apple, arugula, cucumber, and strawberry. Surface roughness varied significantly among produce types (2.51–5.86 µm), with arugula exhibiting the highest values. Hydrophobicity assessments revealed discrepancies between qualitative (contact angle-based) and quantitative (free energy-based) methods: while all produce were classified as hydrophobic qualitatively, strawberry was hydrophilic by quantitative analysis. All bacterial species tested were hydrophilic qualitatively, but *E. coli* showed hydrophobic character quantitatively. Thermodynamic predictions of adhesion (Δ*G_adhesion_*) did not predict observed adhesion bacterial counts (5.07–6.20 log CFU·g^−1^), with substantial bacterial attachment occurring even when thermodynamically unfavorable (positive Δ*G_adhesion_*), indicating that biological factors override physicochemical interactions. Temperature deeply influenced adhesion, with 25 °C promoting 0.3–3.5 log CFU·g^−1^-higher bacterial counts than 7 °C across all combinations (*p*-value ≤ 0.05). These findings demonstrate that bacterial adhesion to fresh produce is multifactorial, with temperature as the dominant controllable factor, and highlight the need for integrated sanitation approaches combining physical and chemical treatments applied before refrigerated storage.

## 1. Introduction

Despite advances in production and handling practices, bacterial contamination of fresh produce remains a persistent global food safety concern. Ready-to-eat (RTE) or minimally processed fruits/vegetables (MPFV) can be colonized by a wide variety of microorganisms, including bacteria, yeasts, and filamentous fungi, which can cause deterioration or food poisoning [1]. The presence of foodborne pathogens in fresh produce has been identified worldwide. Various surveillance studies have consistently demonstrated the widespread occurrence of bacterial pathogens in fresh produce across different geographical regions and product types, including RTE and MPFV [2,3,4,5,6,7].

In parallel, foodborne outbreaks have been reported after the consumption of contaminated fresh produce [8,9]. Contamination rates in fresh produce vary widely by geographical location and product type. However, *Salmonella*, *E. coli*, and *S. aureus* are common pathogens found in these types of products. *Salmonella* is a Gram-negative bacterium that is extensively present in the environment that causes diseases and symptoms are diarrhea, fever, and abdominal pain [10]. Although there are strains of *E. coli* that are harmless to humans, certain species are disease causes, being an important foodborne pathogen that has been reported to contaminate various products. *E. coli* is a facultative anaerobic Gram-negative bacterium, and infection typically causes diarrhea, abdominal pain, fever, nausea, and vomiting [11]. *S. aureus* is one of the most relevant pathogens regarding foodborne illnesses, potentially causing a wide spectrum of diseases in humans. It is a facultative anaerobic Gram-positive bacterium with pathogenicity associated with the production of coagulase enzyme and endotoxin [12]. These observations underscore the global nature of this food safety challenge. While these surveillance studies and outbreak reports effectively document the occurrence and prevalence of pathogen contamination in fresh produce, they offer limited insight into the mechanisms behind pathogen adhesion to produce surfaces. Understanding the physicochemical surface properties that influence initial bacterial attachment is crucial because this step is a prerequisite for colonization, biofilm formation, and subsequent persistence throughout the supply chain. Once attached, bacteria can multiply rapidly when environmental conditions are favorable, including elevated temperatures, high surface moisture, and nutrient availability from plant exudates, becoming progressively more difficult to remove through conventional washing and sanitization procedures [13,14,15].

The process of microbial adhesion to surfaces is a complex phenomenon influenced by multiple factors operating at different scales. Metabolic diversity and adaptation mechanisms to environmental stress are fundamental characteristics of microorganisms that enable them to adhere to various surfaces [16]. At the macroscopic level, environmental conditions such as temperature, pH, ionic strength, and nutrient availability play crucial roles in determining whether adhesion occurs. At the microscopic level, both microbial characteristics (hydrophobicity, surface charge, presence of flagella, fimbriae, and exopolysaccharides) and surface properties (roughness, hydrophobicity, surface charge, and chemical composition) can either favor or inhibit bacterial attachment [17].

Bacterial adhesion to abiotic surfaces, like plastics and metals, or biotic surfaces, like animal or plant cells and tissues, is the initial step for biofilm formation [17,18]. It is considered a very complex process, but generally, on biotic surfaces, it involves molecular interactions mediated by specific receptor–ligand bonds [17]. Biofilms pose a challenge for the food industry because, when not removed, they can make cleaning procedures difficult, cause quality loss, and jeopardize food safety and consumers’ safety [13,14].

The correlation between surface properties such as hydrophobicity and roughness and the adhesion process of microorganisms has been studied on abiotic surfaces (e.g., food processing equipment) [18,19]. However, the influence of these physicochemical characteristics on the adhesion of pathogens to fresh produce surfaces, which have unique and variable biotic properties, remains largely unexplored. This represents a significant research gap for food safety and postharvest science. Understanding the surface properties of foods and microorganisms, and their interactions, is essential for improving our understanding of the adhesion process, selecting more effective sanitizing treatments, and enhancing washing and decontamination procedures, thereby increasing microbiological safety.

Therefore, the present study evaluated the influence of physicochemical factors (roughness, hydrophobicity, the free energy of hydrophobic interaction, the free energy of adhesion, and temperature) on initial microbial adhesion of three major foodborne pathogens (*Salmonella enterica* Enteritidis, *Escherichia coli*, and *Staphylococcus aureus*) to the surface of four commonly consumed fresh produce items (apple, arugula, cucumber, and strawberry).

## 2. Materials and Methods

### 2.1. Experimental Design and Sample Selection

The experiment was conducted in a completely randomized design with three independent biological replicates carried out in different moments.

Fresh produce samples included a whole apple (*Malus domestica* B., var. Gala), arugula leaves (*Eruca sativa* L.), a whole cucumber (*Cucumis sativus* L., var. Aodai), and whole strawberries (*Fragaria × ananassa* Duch, var. Camarosa). The selection of apple, arugula, cucumber, and strawberry were chosen based on high consumption rates, distinct surface characteristics, and limited existing data on bacterial adhesion mechanisms on these surfaces. These vegetables and fruits present distinct surface characteristics, which are known to influence adhesion behavior in fresh produce. Apples are smooth and waxy fruits that generally exhibit lower retention of particles, whereas leafy vegetables like arugula present highly irregular surfaces with veins, trichomes, and microcavities. Strawberries contain natural depressions and achenes that increase the likelihood of contaminant retention, while cucumbers have a relatively smooth yet hydrophilic surface.

Samples were obtained from local retail establishments (Vitória, Espírito Santo, Brazil). Selection criteria included uniform size and absence of visible defects or damage. Upon arrival at the laboratory, samples were gently washed under running tap water to remove visible soil and debris, then air-dried at room temperature (25 ± 2 °C). Samples were used immediately after preparation to minimize changes in surface properties.

### 2.2. Bacterial Strains and Culture Preparation

Three bacterial strains with documented significance in fresh produce-associated foodborne outbreaks were used: *Salmonella enterica* subsp. enterica serovar Enteritidis ATCC 13076, *Escherichia coli* ATCC 11229, and *Staphylococcus aureus* ATCC 25923. Stock cultures were maintained at −80 °C in brain heart infusion (BHI) broth (Difco™, Becton Dickinson, Franklin Lakes, NJ, USA). They were obtained from the culture collection of the Food Hygiene and Microbiology Laboratory at the Federal University of Viçosa (Viçosa, Minas Gerais, Brazil).

For experimental use, cultures were activated by two consecutive transfers into 10 mL of BHI broth (inoculum) and then incubated at 37 °C for 24 h to reach a population of 10^8^–10^9^ CFU·mL^−1^.

### 2.3. Surface Roughness Characterization

The surface microtopography of the samples was evaluated using a 3D optical profilometer (Contour GT-K, Bruker Corporation, Billerica, MA, USA) at the Nanoscopy Laboratory, Physics Department, Federal University of Viçosa (Viçosa, Minas Gerais, Brazil). For each produce type, ten 1 cm^2^ sections were excised from different anatomical regions representative of the edible surface using a sterile scalpel. Average roughness (Ra) values were expressed in micrometers (µm).

### 2.4. Contact Angle Measurements of Fresh Produce and Bacteria

Contact angle measurements of fresh produce samples were performed at 25 ± 1 °C using the sessile drop method [17]. Three probe liquids with different polarities were used: deionized water, formamide (≥99.5% purity, Vetec^®^, Rio de Janeiro, Brazil), and α-bromonaphthalene (≥99% purity, Vetec^®^).

Fresh produce surfaces were prepared by excising 2 × 2 cm^2^ sections from representative areas of samples. A 2.0 µL droplet of each test liquid was gently deposited onto the surface, and the contact angle was measured at 1 s intervals for 15 s, using a contact angle goniometer (DSA100, Krüss GmbH, Hamburg, Germany) equipped with drop shape analysis software. The evaluation was performed in triplicate.

The contact angle of the bacterial cell surface was measured on a layer of vegetative cells using the droplet method described previously [20]. Bacterial cultures were grown in BHI broth to obtain a suspension of active cultures with approximately 1.0 × 10^7^ CFU·mL^−1^. Subsequently, the suspension was centrifuged (4000× *g*, 4 °C, 10 min), washed three times with sterile 0.1 M phosphate-buffered saline (PBS, pH 7.2) to remove growth medium components, and resuspended in PBS.

Cell suspensions were filtered through cellulose acetate membrane filters (0.45 µm pore size, 47 mm diameter, Merck Millipore, Burlington, MA, USA) under vacuum. During the final filtration stage, 30 mL of sterile deionized water (Milli-Q, Darmstadt, Germany) was passed through the filter to remove residual salts that could interfere with contact angle measurements. Filters containing the bacterial layer were immediately transferred to Petri dishes containing solidified agar medium (1% *w*/*v* bacteriological agar supplemented with 10% *v*/*v* glycerol) to maintain consistent moisture content without oversaturation.

Each filter was aseptically divided into three sections, and contact angle measurements were performed on each section using the three probe liquids (water, formamide, α-bromonaphthalene) as described previously [21].

### 2.5. Qualitative Evaluation of Surface Hydrophobicity

Surfaces were qualitatively classified as hydrophilic or hydrophobic based on water contact angle (*θ_w_*) according to the criterion proposed by Vogler [22]: surfaces with *θ_w_* < 65° were classified as hydrophilic, while those with *θ_w_* > 65° were considered hydrophobic.

### 2.6. Determination of the Total Interfacial Tension

The total interfacial tension was determined by the sum of the apolar and polar components of the respective surfaces, according to Equation (1) [21].

(1) $$\gamma_{l}^{TOT}\left(1+cos\;\theta \right)=2\sqrt{\gamma_{s}^{LW}\gamma_{l}^{LW}}+2\sqrt{\gamma_{s}^{-}\gamma_{l}^{+}}+2\sqrt{\gamma_{s}^{+}\gamma_{l}^{-}}$$

where $\gamma_{l}^{TOT}$ is the total interfacial tension of the liquid; $\gamma^{LW}$ is the interfacial tension of the interactions of the Lifshitz–van der Waals forces; $\gamma^{-}$ is the interfacial tension of the electron donor component of the acid–base component; $\gamma^{+}$ is the interfacial tension of the electron acceptor component of the acid–base component; *θ* is the contact angle; and *s* and *l* indicate surface and liquid, respectively.

The three components of the interfacial tension at the surface were determined from the contact angles of three liquids of different polarities [18], whose interfacial tensions are known (Table 1). The sum of two components ($\gamma_{s}^{LW}$ and $\gamma_{s}^{AB}$), according to Equations (2) and (3), results in total interfacial tension ($\gamma_{s}^{TOT}$) (Equation (4)) [21].

(2) $$\gamma_{s}^{LW}=11.1\;(1+cos\;\theta_{B}{)}^{2}$$

(3) $$\gamma_{s}^{AB}=2(\sqrt{\gamma_{s}^{+}\gamma_{s}^{-}})$$

(4) $$\gamma_{s}^{TOT}=\gamma_{s}^{LW}+\gamma_{s}^{AB}$$

where $\gamma_{s}^{LW}$ is the interfacial tension of the interactions of the Lifshitz–van der Waals forces; *θ_B_* is the contact angle obtained with α-bromonaphthalene; $\gamma_{s}^{AB}$ is the polar component of the Lewis acid–base interaction; $\gamma_{s}^{+}$ is the interfacial tension of the electron acceptor component of the acid–base component; $\gamma_{s}^{-}$ is the interfacial tension of the electron donor component of the acid–base component; and $\gamma_{s}^{TOT}$ is the total interfacial tension of the surface.

### 2.7. Free Energy of Hydrophobic Interaction

The total free energy of interaction (${\Delta G}_{sws}^{TOT}$) among molecules of the surface (*s*) immersed in water (*w*) was calculated by the sum of the apolar (${\Delta G}_{sws}^{LW}$) and polar (${\Delta G}_{sws}^{AB}$) free energies of interaction, according to Equations (5)–(7) [21].

(5) $${\Delta G}_{sws}^{TOT}={\Delta G}_{sws}^{LW}+{\Delta G}_{sws}^{AB}$$

(6) $${\Delta G}_{sws}^{LW}=-2\sqrt{\gamma_{s}^{LW}-\gamma_{w}^{LW}}$$

(7) $${\Delta G}_{sws}^{AB}=-4(\sqrt{\gamma_{s}^{+}\gamma_{s}^{-}}+\sqrt{\gamma_{w}^{+}\gamma_{w}^{-}}-\sqrt{\gamma_{s}^{+}\gamma_{w}^{-}}-\sqrt{\gamma_{w}^{+}\gamma_{s}^{-}})$$

### 2.8. Determination of the Total Free Energy of Adhesion

From the values of the interfacial tension components, the total free energy of adhesion (Δ*G_adhesion_*) between the surfaces of the microorganism (*b*) and the food (*s*) was determined according to Equations (8)–(10) [21].

(8) $$\gamma_{bs}=\gamma_{bs}^{LW}+\gamma_{bs}^{AB}$$

(9) $$\gamma_{bs}^{LW}=\gamma_{b}^{LW}+\gamma_{s}^{LW}-2\sqrt{\gamma_{b}^{LW}\gamma_{s}^{LW}}$$

(10) $$\gamma_{bs}^{AB}=2(\sqrt{\gamma_{b}^{+}\gamma_{b}^{-}}+\sqrt{\gamma_{s}^{+}\gamma_{s}^{-}}-\sqrt{\gamma_{b}^{+}\gamma_{s}^{-}}-\sqrt{\gamma_{b}^{-}\gamma_{s}^{+}})$$

Considering that free energy is directly related to the interfacial tension, Δ*G_adhesion_* can be represented as Equations (11)–(13) [21].

(11) $${\Delta G}_{adhesion}={\Delta G}_{bls}^{LW}+{\Delta G}_{bls}^{AB}$$

(12) $${\Delta G}_{bls}^{LW}=\gamma_{bs}^{LW}-\gamma_{bl}^{LW}-\gamma_{sl}^{LW}$$

(13) $${\Delta G}_{bls}^{AB}=\gamma_{bs}^{AB}-\gamma_{bl}^{AB}-\gamma_{sl}^{AB}$$

where $\gamma_{bs}$ is the interfacial tension between the bacterial surfaces and the adhesion surface; *γ_bl_* is the interfacial tension between the bacterial surfaces and the liquid; and *γ_sl_* is the interfacial tension between the adhesion surfaces and the liquid.

The value of Δ*G_adhesion_* allows for a thermodynamic evaluation of the adhesion process, which is thermodynamically favorable when Δ*G_adhesion_* < 0 and unfavorable when Δ*G_adhesion_* > 0.

### 2.9. Bacterial Adhesion Assay

#### 2.9.1. Inoculum Preparation

Working cultures prepared as described in Section 2.2 were standardized to achieve a final concentration of approximately 10^8^–10^9^ CFU·mL^−1^.

#### 2.9.2. Produce Inoculation and Contact Phase

Fresh produce samples were aseptically transferred to individual sterile plastic bags: 200 g of cucumber (the approximate weight of an average cucumber), 100 g of strawberries, 100 g of apple, and 100 g of arugula. To each bag, 10 mL of standardized bacterial inoculum and 1000 mL of sterile 0.1% peptone water were added.

Bags were sealed and gently massaged by hand for 3 min to ensure uniform distribution of bacteria across all produce surfaces. Samples were then maintained in contact with the inoculum suspension for 60 min at 25 ± 2 °C without agitation to allow initial bacterial attachment. The inoculation suspension was subsequently drained aseptically [21,22].

#### 2.9.3. Adhesion Incubation Period

After inoculation and rinsing, contaminated produce samples were transferred to sterile plastic bags and incubated at 25 ± 2 °C (ambient temperature) and 7 ± 1 °C (refrigeration temperature) to simulate different storage conditions. For apple, cucumber, and strawberry, the incubation period was 24 h to allow sufficient time for bacterial adaptation and stabilization of adhesion on the produce surface [23].

For arugula leaves, the incubation period was reduced to 60 min at each temperature due to the rapid deterioration of leafy tissue under wet conditions and preliminary experiments showing that significant bacterial adhesion occurred within this shorter time [23].

Each combination of produce type, bacterial species, and temperature condition was tested in triplicate using independent biological replicates.

#### 2.9.4. Enumeration of Adhered Bacteria

After the incubation period, 10 g of each produce sample was aseptically transferred to sterile plastic bags containing 90 mL of 0.1% sterile peptone water. Samples were homogenized in a stomacher for 2 min at normal speed to detach surface-adhered bacteria. Appropriate serial dilutions were prepared in 0.1% peptone water, and the aliquots were *surface*-plated onto selective *agar* media: *Salmonella*-*Shigella* agar (Difco, Sparks, Maryland, EUA) for *S. enterica*, MacConkey agar (Difco™) for *E. coli*, and Baird-Parker agar (Difco™, Sparks, MD, USA) for *S. aureus*.

Plates were incubated at 37 °C for 24 h, and characteristic colonies were enumerated. Results were expressed as log CFU·g^−1^ of produce.

### 2.10. Statistical Analysis

All experiments were performed with three independent replicates, and data are presented as mean ± standard deviation. Surface roughness data were analyzed by one-way analysis of variance (ANOVA), followed by Tukey’s post hoc test. The effects of temperature on bacterial adhesion were evaluated using two-way ANOVA, and differences between means were identified using Tukey’s post hoc test. Statistical significance was set at α = 0.05 for all analyses and analyses were conducted using SAS On Demand for Academics software online (SAS Institute Inc., Cary, NC, USA).

## 3. Results

### 3.1. Surface Roughness of Fresh Produce

The surface roughness of the four produce types differed significantly (*p* ≤ 0.05) (Table 2). Arugula exhibited the highest average roughness, which was more than twice that of the smoothest surface tested, the apple. Cucumber presented intermediate-high roughness, while strawberry showed intermediate roughness, compared to other samples.

### 3.2. Hydrophobicity Characteristics

#### 3.2.1. Qualitative Hydrophobicity Assessment

The qualitative assessment was performed using the sessile drop method. Contact angles with water (*θ_w_*), formamide (*θ_F_*), and α-bromonaphthalene (*θ_B_*) for all surfaces tested are presented in Table 3.

Using the qualitative classification criterion (*θ_w_* < 65° = hydrophilic; *θ_w_* > 65° = hydrophobic) [19], apple, strawberry, cucumber, and arugula were classified as hydrophobic surfaces. In contrast, all three bacterial species were classified as hydrophilic, with *E. coli* showing the lowest *θ_w_*, followed by *S. enterica* and *S. aureus*.

#### 3.2.2. Quantitative Hydrophobicity and Free Energy of Interaction

The quantitative assessment of hydrophobicity, based on total free energy of interaction (Δ*G_sws_^TOT^*), revealed different classifications compared to the qualitative method (Table 4).

Surfaces exhibiting negative Δ*G_sws_^TOT^* values (indicating hydrophobic character) included apple, cucumber, arugula, and *E. coli*. Conversely, surfaces with positive Δ*G_sws_^TOT^* values (indicating hydrophilic character) included *S. enterica*, *S. aureus*, and strawberry.

The nonpolar (Lifshitz–van der Waals) component (Δ*G_sws_^LW^*) was negative for all surfaces, ranging from −0.17 mJ/m^2^ (*S. enterica*) to −5.04 mJ/m^2^ (strawberry). The polar (acid-base) component (Δ*G_sws_^AB^*) varied considerably, from strongly negative values for cucumber (−72.75 mJ/m^2^) and apple (−60.96 mJ/m^2^) to positive values for *S. aureus* (34.31 mJ/m^2^) and *S. enterica* (33.39 mJ/m^2^).

### 3.3. Thermodynamic Analysis of Bacterial Adhesion

The free energy of adhesion (Δ*G_adhesion_*) and the corresponding adhesion levels (log CFU·g^−1^) varied substantially across the different bacteria–food combinations (Table 5). Negative Δ*G_adhesion_* values indicate thermodynamically favorable adhesion, whereas positive values denote unfavorable interactions.

For *E. coli*, adhesion was predicted to be thermodynamically favorable on arugula, apple, and cucumber. Similarly, *S. aureus* exhibited favorable adhesion on arugula, apple, and cucumber. For *S. enterica*, favorable adhesion was observed on apple and cucumber.

In contrast, thermodynamically unfavorable adhesion (Δ*G_adhesion_* > 0) was predicted for *S. enterica* on arugula and strawberry; for *E. coli* on strawberry; and for *S. aureus* on strawberry.

**Figure 1:**
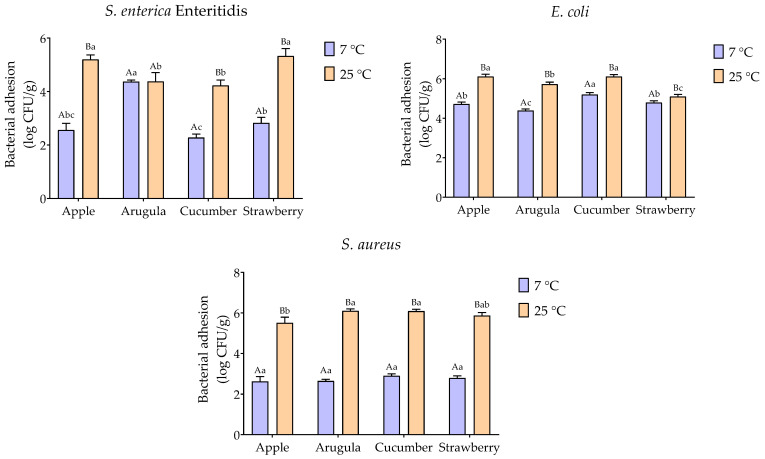
Influence of storage temperature on bacterial adhesion to fresh produce surfaces. Bars represent mean bacterial counts (log CFU·g^−1^) ± standard deviation of triplicate. Different lowercase letters indicate significant differences among produce types at the same temperature (*p* < 0.05, Tukey’s test). Different uppercase letters indicate significant differences between temperatures for the same produce type (*p* < 0.05, Tukey’s test).

Observed bacterial adhesion levels ranged from 5.07 to 6.20 log CFU·g^−1^ across all bacteria-produce combinations tested (Figure 1). Despite the wide variation in predicted thermodynamic favorability (Δ*G_adhesion_* ranging from −46.57 to +29.35 mJ/m^2^), actual adhesion levels showed relatively modest variation, with a difference of only 1.13 log CFU·g^−1^ between the highest and lowest values.

For *S. enterica*, adhesion levels were similar across all four produce types, ranging from 5.07 log CFU·g^−1^ (cucumber) to 5.30 log CFU·g^−1^ (arugula), representing a variation of only 0.23 log CFU·g^−1^. Similarly, *S. aureus* adhesion varied from 5.14 log CFU·g^−1^ (arugula) to 6.18 log CFU·g^−1^ (cucumber), a range of 1.04 log CFU·g^−1^. *E. coli* showed the widest variation, from 5.23 log CFU·g^−1^ (strawberry) to 6.20 log CFU·g^−1^ (apple), a difference of 0.97 log CFU·g^−1^.

Among produce types, cucumber had the highest adhesion for *E. coli* and *S. aureus*, while apple exhibited the highest adhesion for *E. coli*.

### 3.4. Effect of Temperature on Bacterial Adhesion

Temperature significantly affected bacterial adhesion for all bacteria-produce combinations tested (*p* ≤ 0.05) (Figure 1). Storage at 25 °C yielded higher bacterial counts than at 7 °C across all combinations, with the magnitude of the temperature effect varying by bacterial type and produce type.

## 4. Discussion

Contamination of fresh produce by bacterial pathogens remains a persistent global food safety challenge. While surveillance studies show varying prevalence rates depending on geographical region and produce type, the consistent detection of multiple pathogens, including *Salmonella* spp., *E. coli*, and *S. aureus*, across diverse settings underscores the widespread nature of this problem. Notably, pathogen profiles vary considerably: some surveillance studies report high *Salmonella* prevalence in specific produce types and regions, while others have detected low or undetectable *Salmonella* levels but significant contamination with other pathogens.

In Vietnam, a large-scale survey of fresh leafy vegetables (*n* = 572) revealed a relatively high prevalence of *Salmonella* spp. (12.9%), with notable serovar diversity, including *S.* Weltevreden, *S.* Derby, *S.* Lexington, and *S.* Worthington [3]. The detection of multiple serovars suggests diverse contamination sources and highlights the complexity of controlling *Salmonella* in leafy vegetables. In contrast, a study conducted in the United Arab Emirates reported a substantially lower prevalence of *Salmonella* sp. (1.25%) in fresh salad vegetables (*n* = 400) [7].

Additionally, some studies have reported low or undetectable levels of *Salmonella* in MPFV and RTE fresh produce, while still identifying other foodborne pathogens. In Brazil, an analysis of MPFV (*n* = 40) did not detect *Salmonella* sp., although *E. coli* was found in 10% of samples, suggesting inadequate hygiene practices [4]. Similarly, a study conducted in China on fresh-cut fruits and vegetables (*n* = 326) reported the presence of *S. aureus* (15.4%), *E. coli* (9.2%), and *L. monocytogenes* (1.9%), while *Salmonella* sp. was not detected [5]. Large-scale surveillance data from Canada further support these findings, showing a low prevalence of *L. monocytogenes* in RTE fresh-cut fruits (0.51%) and vegetables (0.24%) across 10,070 samples, with no detection of *Salmonella* sp. or *E. coli* O157:H7 [6]. Collectively, these studies highlight that, even in the absence of *Salmonella*, fresh produce may harbor other pathogenic or indicator microorganisms, reinforcing the need for comprehensive microbial risk assessment beyond a single target pathogen.

### 4.1. Surface Roughness as a Determinant of Bacterial Attachment Sites

Among the physicochemical factors that may influence bacterial persistence, surface roughness has been studied as a key factor influencing bacterial adhesion to food surfaces [17,24,25]. The present study evaluated four fresh produce items, including leafy vegetables (arugula), fruiting vegetables (cucumber), and fruits (apple and strawberry). The significant variation in surface roughness among the four fresh produce types tested in the present study reflects their distinct anatomical structures and surface characteristics. Given the roughness of the evaluated fresh produce, it can be expected that, in the present study, the level of microorganism adhesion will increase with increasing roughness.

Higher roughness may favor greater bacterial adhesion to the surfaces of fruits and vegetables. Although not always visible to the naked eye, some foods have rough surfaces and a complex topography formed by undulations and valleys. Arugula, as a leafy vegetable, exhibited the highest roughness, which can be attributed to the complex topography of leaf surfaces, including trichomes, stomata, veins, and intercellular spaces [26].

Wang et al. [24] demonstrated a positive linear correlation between surface roughness and adhesion rate of *E. coli* O157:H7 on various produce surfaces, including apples, navel oranges, avocados, and cantaloupes, supporting the general principle that rougher surfaces promote greater bacterial attachment. The mechanism underlying this phenomenon involves the creation of protected microenvironments within surface depressions and valleys, favoring bacterial fixation and colonization, where bacteria are shielded from shear forces during washing and can access accumulated moisture and nutrients [18,27,28].

However, in the present study, despite arugula having the highest roughness, the observed adhesion levels across different produce types were relatively similar (Table 5), suggesting that roughness alone does not fully determine adhesion outcomes. This observation aligns with findings by Palma-Salgado et al. [25], who reported that increased fruit surface roughness enhanced bacterial adhesion but also reduced decontamination efficiency, indicating a complex interplay between surface topography and other physicochemical factors.

The protective effect of surface irregularities extends beyond simple mechanical shelter. Depressions and elevations on the surface facilitate irreversible adhesion by increasing the contact area between bacterial cells and the substrate, allowing the formation of multiple attachment points through appendages such as fimbriae and flagella. Furthermore, surface irregularities can entrap organic matter and create localized regions of high nutrient concentration, promoting bacterial colonization and subsequent biofilm development [17,28].

For leafy vegetables like arugula, the complexity is further amplified by the presence of natural openings such as stomata, which serve as entry points for bacterial internalization, a process particularly well-documented for *Salmonella* species [15]. This internalization capability may explain why arugula supported substantial *S. enterica* adhesion despite thermodynamically unfavorable predictions, as internalized bacteria would be recovered during the homogenization step but are fundamentally different from surface-adhered cells.

Bacterial attachment to fresh produce surfaces and internalization are serious issues because they can hinder the effectiveness of sanitizers applied to fruits and vegetables. Surface irregularities physically shield bacteria from sanitizer contact and mechanical removal forces, while internalized bacteria are entirely inaccessible to external treatments. These protective mechanisms fundamentally limit the achievable efficacy of post-harvest interventions, underscoring the need for integrated approaches that combine pre-harvest prevention, harvest hygiene, and produce-specific sanitization protocols tailored to surface topography. The surface roughness variations documented in this study suggest that uniform sanitization treatments may be insufficient. Therefore, effective pathogen control requires adapting intervention strategies to the specific physical characteristics of each produce type.

### 4.2. Hydrophobicity Characteristics of Produce and Bacterial Surfaces

#### 4.2.1. Differences Between Qualitative and Quantitative Hydrophobicity Assessments

A notable finding of this study was the difference between qualitative (contact angle-based) and quantitative (free energy-based, ${\Delta G}_{sws}^{TOT}$) hydrophobicity classifications. While the qualitative method classified all four fresh produce types as hydrophobic, the quantitative approach identified strawberries as hydrophilic. This discrepancy highlights the limitations of using a single arbitrary threshold (*θ_w_* = 65°) for binary classification, as proposed by Vogler [22]. While this binary classification of *θ_w_* provides a simplified framework, it should be noted that surface wettability exists on a continuum, and surfaces with *θ_w_* near 65° may exhibit intermediate behavior.

Strawberry’s contact angle with water (66.0°) was only marginally above the 65° cutoff, placing it in an intermediate zone where surface behavior cannot be adequately captured by binary classification. The quantitative thermodynamic approach, which integrates information from three probe liquids with different polarities and considers both apolar (Lifshitz–van der Waals) and polar (acid-base) interactions, provides a more comprehensive assessment of surface energetics. The positive ${\Delta G}_{sws}^{TOT}$ for strawberry reflects the dominance of polar interactions over nonpolar forces, resulting in net hydrophilic character despite the moderately high water contact angle.

Similarly, *E. coli* was classified as hydrophilic by the qualitative method but hydrophobic by the quantitative approach. This apparent contradiction can be reconciled by considering the amphipathic nature of bacterial cell surfaces [29]. While *E. coli* exhibits a strong affinity for water (low *θ_w_*) due to abundant hydrophilic surface components, the overall free energy balance favors self-aggregation over hydration when considering the full spectrum of surface interactions [30,31]. This amphipathic character is advantageous for bacteria, enabling them to interact favorably with both hydrophilic and hydrophobic surfaces depending on environmental conditions.

#### 4.2.2. Bacterial Surface Hydrophobicity and Strain-Specific Variations

The three bacterial species examined in this study displayed distinct hydrophobicity profiles. The thermodynamic characterization of the bacterial surface is directly related to its physicochemical characteristics, which are determined by the presence of compounds such as lipopolysaccharides (LPS), proteins, and exopolymers. It is important to highlight that these compounds, in turn, may be present in different proportions and vary according to growth conditions and strain variation [18,32].

*E. coli* exhibited the lowest water contact angle (16.9°), consistent with its Gram-negative cell wall structure featuring an outer membrane rich in LPS. These LPS molecules present numerous hydrophilic sugar residues and phosphate groups that interact strongly with water [30]. However, the presence of lipid A anchors within the LPS structure contributes hydrophobic character, explaining the negative Δ*G_sws_^TOT^* despite the low *θ_w_* [31].

*S. aureus* and *S. enterica* both demonstrated hydrophilic character by both classification methods. For *S. aureus*, surface properties are dominated by peptidoglycan and teichoic acids in its Gram-positive cell wall, which present numerous hydroxyl and carboxyl groups [30,31,33]. For *S. enterica*, similar to *E. coli*, the LPS-rich outer membrane confers hydrophilic character, though the specific LPS structure varies between serovars [34,35,36].

Microorganisms have amphipathic character; that is, they exhibit both hydrophobic and hydrophilic properties. This occurs due to the interaction of nonpolar and polar groups present in the microbial wall. The hydrophobicity and electrical charge of the bacterial surface are physicochemical forces involved in the adherence of microorganisms to solid surfaces [29]. Furthermore, the presence of capsules in some bacteria, such as *E. coli*, favors adhesion and changes the hydrophobicity of bacteria, influencing the adhesion capacity [37]. Additionally, factors such as nutrient availability, temperature, pH, and osmotic stress can induce expression of surface appendages (flagella, fimbriae, and pili) and alter extracellular polymeric substance (EPS) production, thereby modifying surface hydrophobicity [38,39]. This phenotypic plasticity enables bacteria to adapt their surface properties to favor adhesion under varying conditions encountered in food processing and storage environments.

According to previous works, the reduced hydrophobicity of *E. coli* O157:H7 on lettuce leaves was associated with the presence of extracellular LPS on the cell surface, suggesting that polysaccharides were an important factor affecting cell hydrophobicity [37]. Additionally, a higher hydrophobicity of *E. coli* O157:H7 was observed in cucumber fruit than in cucumber epidermis, which had a lower polysaccharide content.

Palma-Salgado et al. [25] reported similar water contact angles for *E. coli* K12 (22° ± 7°) and *E. coli* O157:H7 (17.9° ± 0.09°) in the sessile drop method, values consistent with our findings for *E. coli* 11229 (16.9° ± 1.4°), suggesting that hydrophilic character is conserved across different *E. coli* strains despite variations in serotype and pathogenic potential.

The physicochemical properties of the surface strongly influence the adhesion of microorganisms, which tend to adhere more readily to hydrophobic than to hydrophilic surfaces. However, the thermodynamic characterization of bacterial surfaces is further complicated by the heterogeneous distribution of surface components. Bacterial cells are not uniform spheres but possess localized regions of varying hydrophobicity, charge, and functionality. Flagella, for example, often exhibit a hydrophobic character distinct from the cell body [40,41], enabling specific interactions with surfaces that average contact angle measurements may not fully capture. Therefore, adhesion can still occur between a hydrophobic and hydrophilic surface or between two hydrophilic surfaces [34].

#### 4.2.3. Produce Surface Chemistry and Wax Composition

The hydrophobicity of fresh produce surfaces primarily depends on the composition and structure of the cuticle, a lipophilic layer covering the aerial parts of plant organs. Apple’s high hydrophobicity (*θ_w_* = 91.5°, Δ*G_sws_^TOT^* = −61.31 mJ/m^2^) reflects its thick epicuticular wax layer composed predominantly of long-chain aliphatic compounds, including alkanes, fatty acids, and esters. This waxy coating provides a water-repellent barrier that protects against desiccation and pathogen invasion [42,43].

Cucumber also exhibited a strong hydrophobic character (*θ_w_* = 70.5°, Δ*G_sws_^TOT^* = −73.86 mJ/m^2^), attributed to its crystalline wax structures visible on the fruit surface [26,44]. Arugula, despite being a leafy vegetable, showed hydrophobic properties (*θ_w_* = 67.3°, Δ*G_sws_^TOT^* = −40.02 mJ/m^2^), though less pronounced than apple or cucumber, reflecting its thinner cuticle layer compared to fruit surfaces.

Strawberry’s intermediate properties (*θ_w_* = 66.0°, Δ*G_sws_^TOT^* = +21.63 mJ/m^2^) likely result from its surface structure, which includes both waxy cuticle regions and protruding achenes (the actual fruits) surrounded by hydrophilic trichomes [26,45]. This heterogeneous surface presents both hydrophobic and hydrophilic domains, contributing to its intermediate classification.

The physicochemical properties of produce surfaces strongly influence microbial adhesion, with the general expectation that bacteria adhere more readily to hydrophobic surfaces due to reduced energy barriers to displacing the aqueous film covering them [20]. However, as demonstrated in this study, adhesion can occur between surfaces of dissimilar hydrophobicity (e.g., hydrophilic bacteria adhering to hydrophobic produce), indicating that other factors beyond simple hydrophobic interactions govern the adhesion process.

### 4.3. Thermodynamic Predictions Versus Observed Adhesion Outcomes

A notable finding of this study was the substantial discrepancy between thermodynamic predictions (Δ*G_adhesion_*) and experimentally observed bacterial adhesion levels (Table 5). Although Δ*G_adhesion_* values spanned a wide range (–46.57 to +29.35 mJ/m^2^), and arugula showed the highest roughness value (Section 3.1), the measured adhesion varied only modestly (5.07 to 6.20 log CFU·g^−1^, a difference of 1.13 log) across samples. Notably, substantial adhesion occurred even under conditions predicted to be thermodynamically unfavorable, such as *S. aureus* on strawberry.

These discrepancies underscore the inherent constraints of the thermodynamic framework, which relies on macroscopic surface parameters and assumes equilibrium conditions. The theory accounts only for physicochemical interactions (Lifshitz–van der Waals, electrostatic, and acid–base forces), while excluding key biological determinants, such as surface appendages, active cell responses, and extracellular polymer, that play a critical role in real adhesion scenarios [46]. The extracellular carbohydrate complexes produced by *E. coli* O157:H7, for example, provide a physical barrier that protects cells against environmental stress [37]. Additionally, both *Salmonella* and many *E. coli* strains are motile, which may explain their ability to adhere despite unfavorable thermodynamic predictions.

Few studies have systematically compared thermodynamic predictions with observed adhesion on fresh produce. Most research focuses on model systems or non-produce surfaces, like glass or polymers [18,19]. However, some investigations have begun exploring real food matrices. For example, a study [19] investigated the factors involved in the adhesion of *S. enterica* Enteritidis and *E. coli* on green peppers and melons. In contrast to the pattern observed in our work, that study reported higher total adhesion energy values for *E. coli* than for *Salmonella*. Moreover, although our results did not show a clear relationship between surface hydrophobicity and adhesion, their findings indicated that the hydrophobic surface of green peppers was associated with increased adherence of both bacteria. In contrast, the more hydrophilic surface of melons was associated with lower adhesion.

Both bacterial cells and produce surfaces display pronounced micro- and nanoscale heterogeneity in their chemical composition and physicochemical properties. This structural complexity creates localized niches that can facilitate attachment, regardless of the overall thermodynamic predictions. Consequently, as observed in the present study and consistently reported in previous investigations [23,26,37,47], pathogenic bacteria can still adhere to these substrates and initiate biofilm formation.

Thermodynamic calculations predict the equilibrium state but provide no information about the kinetics of reaching that state. Adhesion is a time-dependent process involving reversible and irreversible stages. Initial, reversible adhesion occurs rapidly (seconds to minutes) and is governed primarily by physicochemical forces captured in thermodynamic models. However, irreversible adhesion develops over longer timescales (minutes to hours) as bacteria produce EPS, express specific adhesins, and establish molecular bridges to the surface.

In this study, the contact time before washing (1 h) and subsequent incubation period (24 h for most produce, 60 min for arugula) allowed ample time for irreversible adhesion to develop, potentially explaining why even thermodynamically unfavorable combinations showed substantial adhesion. Bacteria may initially attach weakly or to localized favorable sites, then progressively strengthen their attachment through biological mechanisms that overcome unfavorable bulk thermodynamics.

Despite these limitations, thermodynamic approaches remain highly valuable. They provide a foundational framework for understanding the physicochemical forces that govern early-stage bacterial approach and interaction with surfaces. Such models allow researchers to identify general adhesion trends, compare substrates under standardized criteria, and generate mechanistic hypotheses that can be further validated experimentally. Thus, even though adhesion in real systems is strongly influenced by biological and microscale surface heterogeneity, thermodynamic predictions still constitute an essential component of a comprehensive evaluation of bacterial attachment.

The discrepancy between thermodynamic predictions and biological outcomes underscores the need for integrative models that incorporate both physicochemical and biological variables. From a practical standpoint, the inability of thermodynamic models to reliably predict adhesion outcomes suggests that produce sanitization strategies cannot be based solely on surface hydrophobicity measurements. Instead, surface topography (roughness), biological characteristics of target organisms (motility, adhesin expression), and environmental factors (temperature, contact time, presence of organic matter) must all be considered when designing intervention strategies.

### 4.4. Temperature-Dependent Adhesion Patterns and Storage Implications

The significant enhancement of bacterial adhesion at 25 °C compared to 7 °C across all bacteria-food combinations (Figure 1) reflects the fundamental influence of temperature on microbial metabolism and adhesion kinetics. At 25 °C, room-temperature storage condition, bacterial cells exhibit heightened metabolic activity, enhanced motility, and increased production of adhesion-related structures such as flagella, fimbriae, and EPS.

The most intriguing finding was that *S. enterica* adhesion to arugula showed no significant temperature difference, while adhesion to other produce increased substantially at 25 °C. This unique pattern can be attributed to *Salmonella*’s ability to internalize in leafy vegetables [15]. *Salmonella* spp. can enter plant tissues through stomata, wounds, and cut edges, establishing internal populations that are protected from surface environmental conditions [15]. At 7 °C, while surface adhesion may be limited, internalized bacteria remain viable and contribute to total recovered counts. At 25 °C, both surface and internal populations increase, but the overall effect is moderated by the already-substantial internal population established at lower temperatures.

González-López et al. [47] demonstrated that temperature was the most influential factor for *Salmonella* spp. development on apples, with optimal colonization at 22 °C compared to 5 °C and 15 °C, supporting our findings. However, Grivokostopoulos et al. [15] reported that for *S. enterica* Enteritidis on leafy vegetables (arugula, chicory, lettuce, spinach), increasing storage time from 2 to 48 h had a greater impact on growth than temperature increases from 5 to 20 °C, consistent with our observation that time-dependent internalization may override temperature effects on arugula.

*E. coli* showed consistent temperature-dependent increases across all produce types, with the magnitude varying by substrate. The smallest temperature effect occurred on strawberry, possibly due to strawberry’s intermediate hydrophobicity and high surface heterogeneity, which may provide sufficient microenvironments for adhesion even at suboptimal temperatures. The largest increase was observed in arugula, where the rough, complex surface topology, combined with enhanced bacterial metabolism at 25 °C, facilitated extensive colonization.

Sun et al. [37] investigated *E. coli* O157:H7 adhesion and biofilm formation on cucumber stored at 4 °C and 25 °C, observing greater extracellular material production and biofilm formation at 25 °C, particularly in stomatal tissues. They further noted that biofilm formation capacity varied by tissue type (vascular system > fruit tissue > epidermis), highlighting the importance of surface anatomy in adhesion processes. This factor likely contributes to the observed temperature effects in our study.

Given that MPFV foods can undergo peeling and slicing steps, which expose their nutrients, it is essential to understand the parameters that favor biofilm adhesion and formation.

*S. aureus* exhibited the most pronounced temperature effect, with uniformly low adhesion at 7 °C but substantial increases at 25 °C. This marked temperature dependency reflects *S. aureus*’s growth characteristics as a mesophile with optimal growth between 35–37 °C. At 7 °C, which is near its minimum growth temperature, *S. aureus* metabolism is severely suppressed, limiting both growth and adhesion. At 25 °C, metabolic activity increases substantially, enabling robust adhesion and growth.

*S. aureus*’s broad environmental adaptability allows it to survive and multiply across a wide temperature range, though growth rates vary significantly [48]. The consistent temperature effect across all produce types suggests that temperature acts as a master regulator of *S. aureus* adhesion, potentially overriding substrate-specific differences that might be more prominent at optimal growth temperatures. However, fresh produce can be contaminated with human pathogens at any stage of processing. Contamination may occur both preharvest and postharvest due to inappropriate practices, including the use of contaminated irrigation and washing water, contaminated soil, the presence of animals, physical damage, and improper handling. Furthermore, cross-contamination can occur during the shredding, cutting, and packaging of fruits and vegetables [4,5].

The reduced but still substantial bacterial adhesion observed for *S. aureus* at 7 °C, depending on the bacteria and the produce, has important implications for fresh produce safety. Storage at low temperatures is one of the methods applied to decrease the growth rate of microorganisms and increase the shelf life of the food [49]. While refrigeration effectively slows bacterial growth, it does not entirely prevent adhesion. Bacteria that successfully adhere under cooler temperatures can persist on produce surfaces, and their populations may increase if temperature control is compromised during distribution or retail display, a common occurrence in fresh produce supply chains.

Moreover, exposure to cold stress can induce adaptive responses in bacteria that enhance survival and stress tolerance, thereby favoring bacterial resistance to sterilization procedures. Lim & Ha [50] demonstrated that *E. coli* O157:H7 and *S. enterica* Typhimurium grown on lettuce at 15 °C exhibited greater resistance to X-ray irradiation (0.2–0.6 kGy) compared to bacteria grown at 25 °C or 37 °C. This phenomenon, termed cross-protection, indicates that suboptimal temperatures may select for hardy bacterial subpopulations with enhanced resistance to subsequent sanitization treatments. For *E. coli*, resistance was highest in lettuce stored at 15 °C, whereas for *S. enterica* Typhimurium, bacterial survival after irradiation was greater at 15 °C than at 25 °C or 37 °C.

These findings suggest a dual effect: although refrigeration slows bacterial growth, it may also favor the survival of cells that become more tolerant to stress and harder to remove. This highlights the need to apply effective sanitization before refrigerated storage to reduce the number of bacteria that undergo cold adaptation.

An integrated approach combining physical and chemical technologies offers the most effective strategy for controlling bacterial adhesion on fresh produce. Physical treatments, such as high-pressure washing or ultrasound, can mechanically remove bacteria from surface irregularities identified in our roughness analysis. At the same time, chemical sanitizers provide antimicrobial activity against remaining cells. The combined effect of these approaches, with physical methods enhancing sanitizer access and chemical agents inactivating mechanically resistant bacteria, addresses the multifactorial nature of adhesion more effectively than single interventions. Critically, these combined treatments should be applied immediately post-harvest, before cooling, to maximize efficacy against metabolically active, non-adapted bacterial populations. This multi-hurdle strategy, coupled with strict cold chain maintenance, represents best practice for minimizing pathogen risks associated with fresh produce.

### 4.5. Conclusions

This study offers a detailed analysis of the physical, chemical, and environmental factors that affect bacterial adhesion to fresh produce surfaces. It shows that adhesion is a complex process influenced by many factors and cannot be predicted by surface properties alone. Although surface roughness varied greatly among produce types and hydrophobicity assessments differed between qualitative and quantitative methods, these factors had limited correlation with actual adhesion levels. Thermodynamic predictions based on free energy calculations sometimes did not align with experimental results, highlighting the importance of biological factors such as bacterial appendages, EPS, and active metabolism over passive physicochemical interactions. Temperature was identified as the most consistent and significant factor influencing adhesion, with 25 °C leading to notably higher bacterial counts than 7 °C across all bacteria-produce combinations. However, the fact that refrigeration cannot completely prevent adhesion underscores its limitations as a sole control measure.

These findings have significant practical implications for fresh produce safety. The reduced predictive value of surface hydrophobicity and thermodynamic models indicates that sanitization strategies cannot depend solely on surface characterization. Instead, they must consider the biological complexity of pathogen adhesion. The notable effects of temperature, along with evidence of cold-adapted stress responses, highlight the importance of timing interventions, particularly performing sanitization immediately after harvest before bacteria form strong attachments and adapt to cold conditions. An integrated approach that combines physical removal (targeting surface roughness barriers to sanitizer penetration) with chemical inactivation (targeting remaining adherent bacteria), should be applied before refrigerated storage to best minimize pathogen risk.

However, it is important to note that this study was performed under a limited set of storage conditions, evaluating only two temperatures. As a result, the conclusions are limited to common fresh produce storage situations and should not be extrapolated to different temperatures, thermal processing, or post-harvest interventions such as washing, sanitization, or cooking, all of which can significantly affect bacterial survival, adhesion, and detachment. In this context, significant knowledge gaps remain regarding the effects of processing-related stresses on microbial attachment and persistence, the relative contributions of specific biological adhesion mechanisms (flagella, fimbriae, EPS production), and the dynamics of bacterial internalization in leafy produce. Addressing these gaps through systematic research will enable the fresh produce industry to move from empirical, generic approaches toward targeted, mechanism-based interventions that more effectively protect public health while maintaining product quality.

## Figures and Tables

**Table 1:** Components of the interfacial tension of liquids at 25 °C.

Liquid	Interfacial Tension (mJ·m^−2^)			
	$\gamma_{s}^{TOT}$	$\gamma_{l}^{LW}$	$\gamma_{l}^{+}$	$\gamma_{l}^{-}$
α-Bromonaphthalene (*θ_B_*)	44.4	44.4	0.0	0.0
Water (*θ_w_*)	72.8	21.8	25.5	25.5
Formamide (*θ_F_*)	58.0	39.0	2.28	39.6

Values of interfacial tension were obtained based on São José et al. [18].

**Table 2:** Surface roughness of fresh produce measured by an optical profilometer.

Sample	Roughness (*Ra*) Means ± Standard Deviation (µm)
Apple	2.51 ± 0.12 ^a^
Arugula	5.86 ± 0.11 ^d^
Cucumber	4.35 ± 0.18 ^c^
Strawberry	3.09 ± 0.39 ^b^

Means and standard deviations with different superscript letters differ significantly by Tukey’s test (*p* ≤ 0.05).

**Table 3:** Contact angle values (mean ± standard deviation) with water (*θ_w_*), formamide (*θ_F_*), and α-bromonaphthalene (*θ_B_*) on different surfaces.

Surface	Contact Angle (°)		
	*θ_w_*	*θ_F_*	*θ_B_*
*E. coli*	16.9 ± 1.4	55.5 ± 4.5	46.1± 3.5
*S. aureus*	24.6 ± 4.3	24.9 ± 0.9	29.6 ± 3.6
*S. enterica*	21.2 ± 4.0	32.1 ± 2.6	60.6 ± 3.4
Apple	91.5 ± 4.4	66.5 ± 2.6	58.3 ± 4.3
Arugula	67.3 ± 7.4	66.8 ± 8.3	41.0 ± 5.1
Cucumber	70.5 ± 5.9	70.9 ± 3.0	51.3 ± 2.3
Strawberry	66.0 ± 5.4	71.7 ± 2.7	27.9 ± 5.0

Surfaces with *θ_w_* < 65° are classified as hydrophilic, while *θ_w_* > 65° indicates hydrophobic character according to Vogler [22].

**Table 4:** Nonpolar (Δ*G_sws_^LW^*), polar (Δ*G_sws_^AB^*), and total free energy of interaction (Δ*G_sws_^TOT^*) components of different surfaces.

Sample	Δ*G_sws_^LW^* (mJ/m^2^)	Δ*G_sws_^AB^* (mJ/m^2^)	Δ*G_sws_^TOT^* (mJ/m^2^)
*E. coli*	−1.89	−19.72	−21.61
*S. enterica*	−0.17	33.39	33.21
*S. aureus*	−4.87	34.31	29.45
Apple	−0.35	−60.96	−61.31
Arugula	−2.32	−37.70	−40.02
Cucumber	−1.11	−72.75	−73.86
Strawberry	−5.04	26.67	21.63

Surfaces with negative Δ*G_sws_^TOT^* are classified as hydrophobic, while positive values indicate hydrophilic character.

**Table 5:** Free energy of adhesion (Δ*G_adhesion_*, mJ/m^2^) and observed bacterial adhesion levels (log CFU·g^−1^) for different bacteria-produce combinations.

Bacteria × Surface	Δ*G_adhesion_* (mJ/m^2^)	Adhesion (log CFU·g^−1^)
*S. enterica* × Arugula	5.11	5.30
*S. enterica* × Strawberry	29.35	5.20
*S. enterica* × Apple	−5.24	5.12
*S. enterica* × Cucumber	−3.44	5.07
*E. coli* × Arugula	−31.43	5.84
*E. coli* × Strawberry	5.71	5.23
*E. coli* × Apple	−43.28	6.20
*E. coli* × Cucumber	−46.57	6.13
*S. aureus* × Arugula	−5.47	5.14
*S. aureus* × Strawberry	25.33	5.92
*S. aureus* × Apple	−16.69	5.53
*S. aureus* × Cucumber	−16.54	6.18

Negative Δ*G_adhesion_* values indicate thermodynamically favorable adhesion, while positive values indicate unfavorable adhesion. CFU: colony-forming units.

## Data Availability

The datasets presented in this article are not readily available due to technical limitations. Requests to access the datasets should be directed to the authors.

## Abbreviations

The following abbreviations are used in this manuscript:

BHI	Brain heart infusion
EPS	Extracellular polymeric substance
MPFV	Minimally processed fruits and vegetables
PBS	Phosphate-buffered saline
RTE	Ready-to-eat
